# Systematic review with network meta-analysis: comparative efficacy of different enteral immunonutrition formulas in patients underwent gastrectomy

**DOI:** 10.18632/oncotarget.15580

**Published:** 2017-02-21

**Authors:** Guo-Min Song, Xiao-Ling Liu, Wei Bian, Jing Wu, Yong-Hong Deng, Hui Zhang, Xu Tian

**Affiliations:** ^1^ Department of Nursing, Tianjin Hospital, Tianjin 300211, China; ^2^ Department of Gastroenterology, Chongqing Cancer Institute and Hospital and Cancer Center, Chongqing 400030, China; ^3^ Ophthalmology Department, Southwest Hospital, Third Military Medical University, Chongqing 400031, China; ^4^ Graduate College, Tianjin University of Traditional Chinese Medicine, Tianjin 300193, China

**Keywords:** stomach neoplasm, surgical resection, enteral nutrition, immunonutrition, meta-analysis

## Abstract

**Objectives:**

Optimal enteral immunonutrition (EIN) regime for gastric cancer (GC) patients underwent gastrectomy remains uncertainty. To assess comparative efficacy of different EIN formulas in GC patients underwent gastrectomy, we performed network meta-analysis.

**Results:**

We included 11 RCTs enrolling 840 patients. Pairwise meta-analysis indicated that EIN (RR 0.56, 95% CI 0.36-0.86; MD -0.42, 95% CI -0.74—0.10), Arg+RNA+ω-3-FAs (RR 0.37, 95% CI 0.22-0.63; MD -0.42, 95% CI -0.75—0.07), Arg+Gln+ω-3-FAs (RR 0.22, 95% CI 0.05-0.94; MD -0.69, 95% CI -1.22—1.07) reduced ICs and LOS. Network meta-analysis confirmed the potential of Arg+RNA+ω-3-FAs for ICs (OR 0.27, 95% Crl 0.12–0.49) and Arg+Gln+ω-3-FAs for CIs (OR 0.22, 95% Crl 0.02–0.84) and LOS (SMD -0.63, 95% Crl -1.07—0.13), and indicated that Arg+RNA+ω-3-FAs was superior to Arg+RNA and Arg+Gln for ICs as well.

**Materials and Methods:**

We performed direct and network meta-analyses for randomized controlled trials comparing EIN formulas with each other or standard enteral nutrition (SEN) in reducing infectious complications (ICs), noninfectious complications (NICs) and length of hospital stay (LOS), through January 2016. The surface under the cumulative ranking curve (SCURA) and Grading of Recommendations Assessment, Development and Evaluation (GRADE) were used to rank regimes and rate qualities of evidences respectively.

**Conclusions:**

As for GC patients underwent gastrectomy, Arg+RNA+ω-3-FAs and Arg+Gln+ω-3-FAs are the optimal regimes of reducing ICs and LOS.

## INTRODUCTION

Gastric cancer (GC) is the one of the most common digestive tract cancers, which is the fifth most common type of cancer and the third most cause of cancer-death [1]. Issued data estimated that GC will annually cause 989 and 738 thousands new cases and cancer deaths respectively around the world [2]. Surgical resection is still a promising treatment option of curatively treating the GC patients [3]; however, GC patients underwent gastrectomy are at high risk of suffering a variety of complications, such as postoperative infectious complications, non-infectious complications and immunity suppression [4–6].

In purpose of improving the immune function, relieving the inflammation response, and decreasing the postoperative complications of the GC patients underwent gastrectomy, as well as shortening the length of hospital stay (LOS) eventually, enteral immunonutrition (EIN) which is supplemented with at least 2 of arginine (Arg), glutamine (Gln), omega-3 fatty acids (ω-3-FAs), and ribonucleic acid (RNA) has been extensively investigated in clinical practice [4, 7, 8]. Our previous meta-analysis [4] demonstrated the potential of EIN in improving host immunity and decreasing the inflammation response of GC patients underwent gastrectomy compared to standard enteral nutrition (SEN). However, the EIN support regime includes various formulas, such as the combination of SEN, Arg and RNA and the combination of SEN, Arg and Gln. Hitherto, no trial has been planned to investigate the comparative efficacy of different EIN formulas. As a result, it is unclear which EIN formulas are the optimal nutrition support regimes for GC patients underwent gastrectomy.

Bayesian network meta-analysis, which is the expansion of pairwise meta-analysis, provides option for investigators to evaluate the comparative efficacies of multiple treatments which are not directly compared in randomized controlled trials (RCTs) [9]. And thus, we performed direct meta-analysis and network meta-analysis combining multiple direct evidences to evaluate the comparative efficacies of different EIN formulas for the support of GC patients underwent gastrectomy in this present study.

## RESULTS

### Study selection

**Figure 1:**
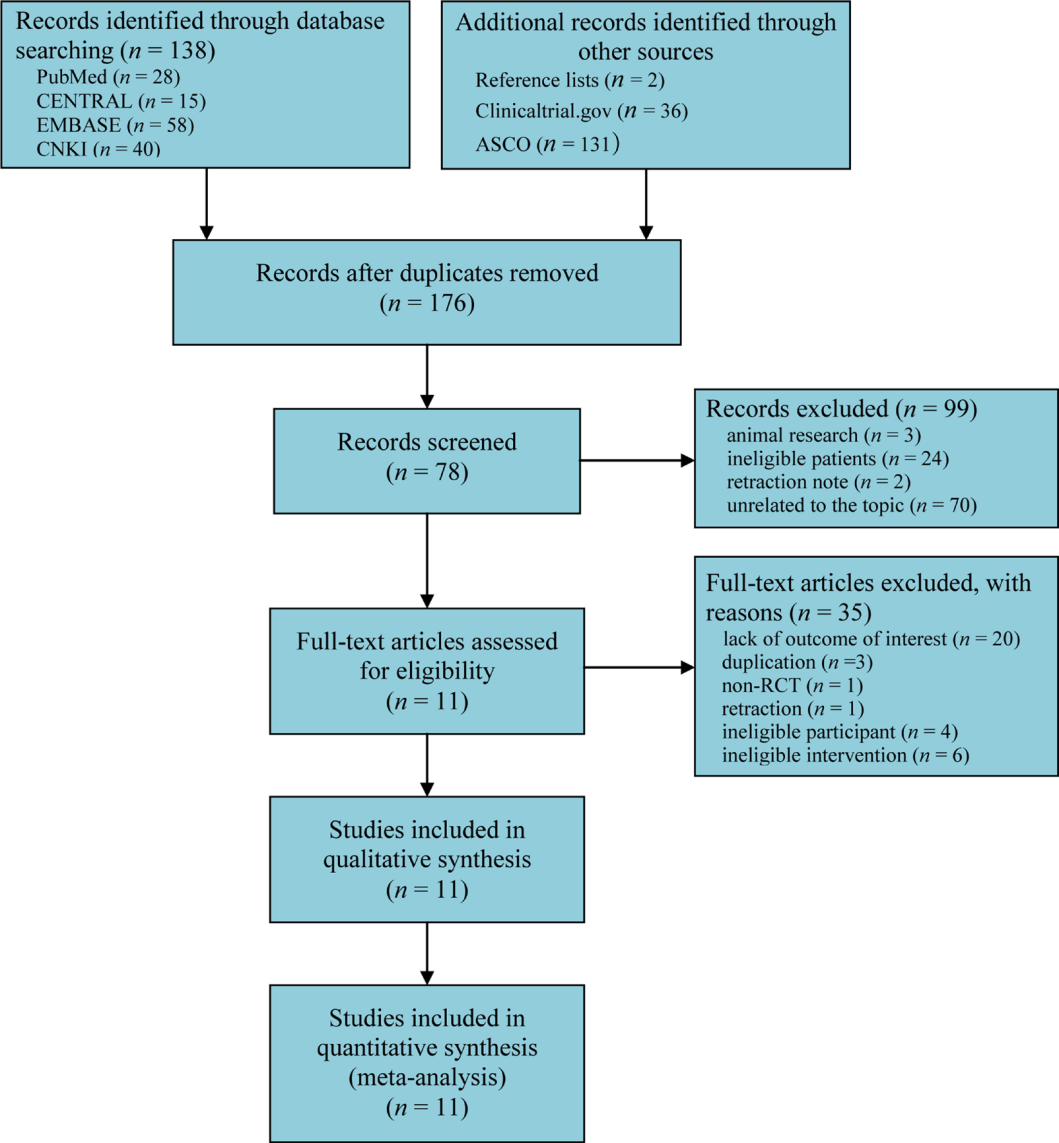
Flow chart of identification and selection of studies CENTRAL = Cochrane Central Register of Controlled Trials, RCTs = randomized controlled trials.

We initially captured 138 records after searching all target databases. One hundred and sixty-nine citations were added through screening reference lists and electronically searched clinicaltrial.gov and American Society of Clinical Oncology (ASCO). One hundred and thirty-one duplicates were omitted by using EndNote software (version X7.0). We excluded 99 records according to following reasons: animal research, ineligible patients, retraction note, and unrelated to topic after screening title and abstract. After examining full-text, 11 studies [3, 6, 10–18] were determined to meet our inclusion criteria. The flow chart of identification and selection of captured studies were summarized in Figure 1.

### Study characteristics

Table 1 documented the details of the characteristics of all eligible studies. Overall, these eligible studies were published between 2005 and 2014, and most were performed in China. Eleven RCTs enrolled 840 patients, and the number of patients in individual study ranged from 42 to 231. Two studies [3, 11] reported the nutrition status of patients. All studies [3, 6, 10–18] reported ICs, 4 [3, 6, 10, 16] reported NICs, and 9 [3, 6, 10–13, 15, 16, 18] reported the postoperative LOS.

**Table 1:** Basic characteristics of each study included in this network meta-analysis

Study ID	Country	Diagnosis	Age of Participants (years)	Number of Participants (T/C)	Nutrition status (Malnutrition/Well nutrition)	Intervention regimes		Reported outcomes
						Treatment group	Control group	
Farreras N 2005[24]	Spain	Gastric cancer	66.7 ± 8.3/ 69.2 ± 13.8	30/30	13/53	Early immune-enhanced nutrition EN given product which enriched with Arg, Gln and ω-3-FAs for the next 7 days	Patients received standard EN support for the next 7 days	ICs, LOS
Fujitani K 2012[3]	Japan	Gastric adenocarcinoma	64 (26~78)/ 65 (30~79)	120/111	239/5	Patients received 1000 ml/day of preoperative oral supplementation in the form of an immunonutrients-enriched enteral feed which mainly composed by Arg of 1.28 g/100 mL and RNA of 0.13 mg/mL) added to normal diet for 5 consecutive days before surgery	Patients received regular diet without any nutritional supplementation for 5 consecutive days before surgery	ICs, NICs, LOS
Liu H 2012[25]	China	Advanced gastric cancer	57.3 ± 7.1/ 58.4 ± 6.3	28/24	Not stated	Patients received standard EN of 500 mL per bottle consisting of 20.0 g total protein, 9.5 g fat, 61.5 g carbohydrate, 7.5 g fiber, 3.0 g minerals and 0.15 g vitamins, providing 500 kcal of total energy supplemented with formula enriched with Gln (12.5 g/L) and Arg (9.0 g/L) for the next 7 days	Patients received standard EN of 500 mL per bottle consisting of 20.0 g total protein, 9.5 g fat, 61.5 g carbohydrate, 7.5 g fiber, 3.0 g minerals and 0.15 g vitamins, providing 500 kcal of total energy for the next 7 days	ICs, LOS
Liu H 2011[26]	China	Advanced gastric cancer	71.5 ± 6.1/ 74.1 ± 9.3	28/28	Not stated	Patients were supplemented with immune nutrition enriched with Arg of 9.0 g/ and Gln of 12.5 g/L in addition to the standard EN for the next 7 days	Patients received standard EN for the next 7 days	ICs, LOS
Liu Z 2011[27]	China	Advanced gastric cancer	61.1 ± 7.5/ 61.6 ± 7.2	21/21	Not stated	Patients were supplemented with immunonutrients enriched with Arg, ω-3-FAs and RNA in addition to the standard EN for the first day after surgery lasted for 8 days	Patients were received standard EN for the first day after surgery lasted for 8 days	ICs
Marano L 2013[6]	Italy	Gastric adenocarcinoma	66.6(55–78)/ 65.1(49–83)	54/55	63/115	Patients received immune nutrition enriched with Arg, ω-3-FAs and RNA in the 6 h after the surgery until the 7 postoperative day	Patients received standard EN in the 6h after the surgery until the 7 postoperative day	ICs, NICs, LOS
Okamoto Y 2009[29]	Japan	Gastric cancer	66.9 ± 11.5/ 70.9 ± 13.2	30/30	Not stated	Patients were given 750 ml per day immune-enhanced formulas supplemented with Arg of 9.6 g, RNA of 0.96, and ω-3 FAs of 3.1 g for 7 consecutive days before the operation.	Patients received isoenergetic standard formulas for 7 consecutive days before the operation.	ICs, NICs, LOS,
Xue JB 2011[31]	China	Gastric cancer	56.6 ± 8.9/ 58.2 ± 8.0	26/26	Not stated	Early immune-enhanced nutrition EN given product which enriched with Arg, Gln and ω-3-FAs for the second day after surgery lasted for 7 days	Patients received standard EN support for the second day after surgery lasted for 7 days	ICs, LOS
Xie Q 2010[30]	China	Gastric cancer	62.5 ± 11.9/ 61.3 ± 11.7	29/29	Not Stated	Patients were supplemented with immunonutrients enriched with Arg, ω-3-FAs and RNA in addition to the standard EN for the first day after surgery lasted for 8 days	Patients were received standard EN for the first day after surgery lasted for 8 days	ICs
Lu QC 2009[28]	China	Gastric cancer	68.6 ± 5.6/ 69.1 ± 5.9	25/25	Not Stated	Patients were supplemented with immunonutrients enriched with Arg, ω-3-FAs and RNA in addition to the standard EN for the second day after surgery lasted for 7 days	Patients were received standard EN for the second day after surgery lasted for 7 days	ICs, LOS
Chen BS 2014[23]	China	Gastric cancer	66.3 ± 8.6/ 66.5 ± 8.2	35/35	Not Stated	Patients were supplemented with immunonutrients enriched with Arg, ω-3-FAs and RNA in addition to the standard EN for the seventh day before surgery lasted the seventh days after 7 surgery	Patients were received standard EN for the seventh day before surgery lasted the seventh days after 7 surgery	ICs, NICs, LOS

T = treatment group, C = control group, EN = enteral nutrition, EIN = enteral immunonutrition, Arg = arginine; Gln = glutamine, RNA = ribonucleic acid, ω-3-FA = omega-3-fatty acids, ICs = infectious complications, NICs = noninfectious complications, LOS = length of hospital stay.

### Quality of individual study

We graphically illustrated the cumulative percentages for each risk of bias domains in Supplementary Figure 1 and risk of bias summary for individual randomized controlled trials in Supplementary Figure 2. Of 11 eligible RCTs, 5 [3, 11, 12, 14, 17] used appropriate method (such as random number table) to generate random sequence, 2 [3, 11] performed appropriately allocation concealment and blinded patients and personnel, only 1 [12] blinded outcome assessor, all [3, 6, 10–18] reported anticipated outcomes and did not selectively reported results.

### Evidence network

**Figure 2:**
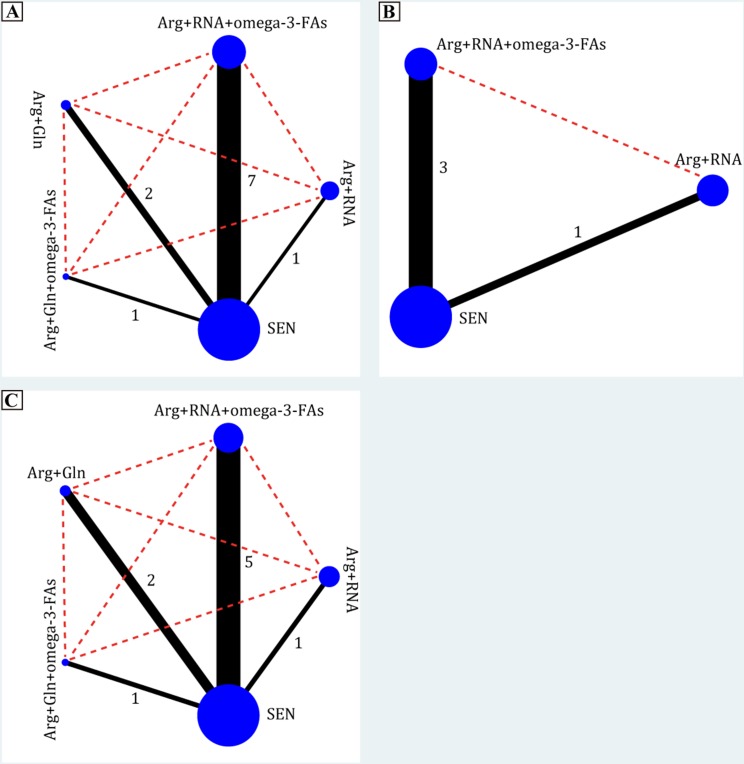
Evidence networks of all enteral immunonutrition formulas in terms of ICs, NICs and LOS The black solid line indicated direct comparisons between regimes which were directly compared in original studies and red dotted line indicated indirect comparisons of two regimes which were not directly compared in original studies. The node and edge was weighted by total sample size and standard error respectively. Arg = arginine, Gln = glutamine, RNA = ribonucleic acid, ω-3-FA = omega-3-fatty acids, ICs = infectious complications, NICs = noninfectious complications, LOS = length of hospital stay.

In the present systematic review and network meta-analysis, we identified 4 EIN formulas: combination of Arg and RNA, combination of Arg, RNA and ω-3-FAs, combination of Arg and Gln and combination of Arg, Gln and ω-3-FAs. All EIN formulas were directly compared with SEN, but no study comparing EIN formulas with each other was identified. Evidence networks in terms of ICs, NICs and LOS were delineated in Figure 2.

### Infectious complications

All 11 eligible RCTs [3, 6, 10–18] reported the ICs, which directly compared EIN formulas with SEN. Compared to SEN, EIN regardless of formulas (11 RCTs, RR 0.56, 95% CI 0.36–0.86), Arg+RNA+ω-3-FAs (7 RCTs, RR 0.37, 95% CI 0.22–0.63), and Arg+Gln+ω-3-FAs (1 RCT, RR 0.22, 95% CI 0.05–0.94), but not Arg+RNA (1 RCT, RR 1.05, 95% CI 0.80–1.39) or Arg+Gln (2 RCTs, RR 0.89, 95% CI 0.53–1.49), decreased the incidence of ICs (see Supplementary Figure 3A).

**Figure 3:**
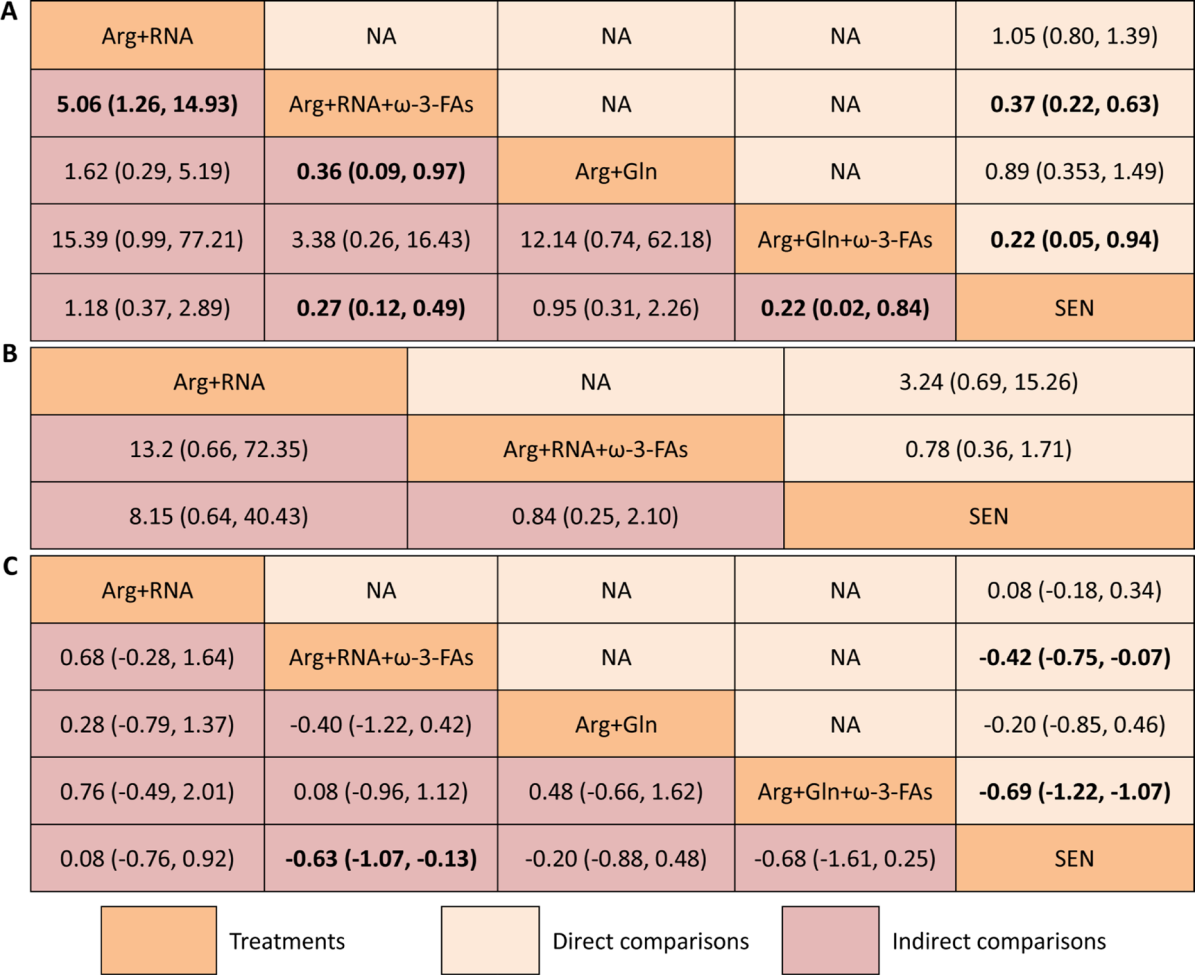
Summary for infectious complications (**A**), non-infectious complications (**B**) and lengths of hospital stay (**C**) of different nutrition support regimes. For categorical data including infectious and non-infectious complications, the upper right area represented the effect sizes of direct comparisons and the bottom left shown the indirect comparisons. For direct comparison, it favors the row-defining treatment if odds ratio (OR) lower than 1, in contrast, for indirect comparison, the result favors the column-defining treatment if OR lower than 1. For numerical data, each number in each cell represented the effect size of the treatment in upper left area minus the treatment in bottom right area. Standard mean differences (SMDs) lower than 0 favor the column-defining treatment. The upper right area presented the effect sizes of direct comparisons and the bottom left shown the direct comparisons. A number with bold font indicated a significant difference between two treatments. SEN: standard enteral nutrition.

In network meta-analysis, Arg+RNA+ω-3-FAs (RR 0.27, 95% CI 0.12–0.49) and Arg+Gln+ω-3-FAs (RR 0.22, 95% CI 0.02–0.84), but not Arg+RNA (RR 1.18, 95% CI 0.37–2.89) or Arg+Gln (RR 0.95, 95% CI 0.31–2.26), were superior to SEN in reducing ICs. Compared to Arg+RNA+ω-3-FAs, Arg+RNA significantly increased the ICs (RR 5.06, 95% CI 1.26–14.93). Arg+RNA+ω-3-FAs was also superior to Arg+Gln in reducing ICs (RR 0.36, 95% CI 0.09–0.97). Remaining comparisons did not indicate significant differences. All estimates from direct and network meta-analysis were summarized in Figure 3A.

**Figure 4:**
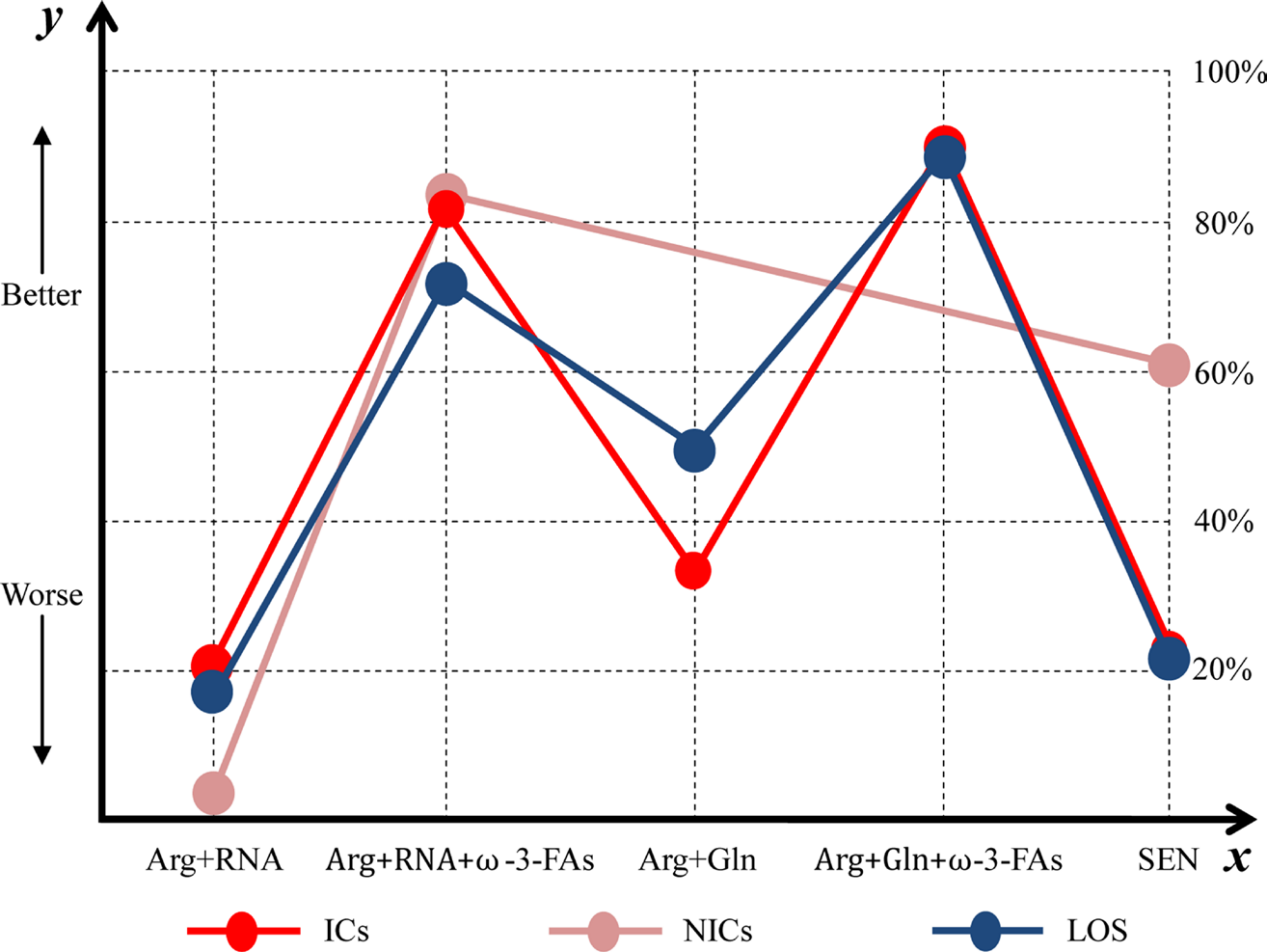
Ranking of all enteral immunonutrition formulas in terms of ICs, NICs and LOS *y* axis represented a treatment will become better option from bottom to top. The percentages which were presented in right vertical dotted line represented the probability of becoming the best efficacious option and *x* axis lists all comparative nutrition support regimes. Arg = arginine, Gln = glutamine, ω-3-FAs = omega-3-fatty-acids, RNA = ribonucleic acid, ICs = infectious complications, LOS = length of hospital stay.

We estimated SUCRA to rank all nutrition support regimes in controlling ICs. The corresponding value of Arg+RNA, Arg+RNA+ω-3-FAs, Arg+Gln, Arg+Gln+ω-3-FAs, and SEN was 21.81%, 81.38%, 33.65%, 90.60%, and 22.56% respectively (see Supplementary Figure 4A). The ranking of all treatments for ICs can be found in Figure 4.

### Non-infectious complications

Of eligible 11 RCTs, 4 [3, 6, 10, 16] directly comparing the EIN formulas with SEN reported the NICs. Direct meta-analysis indicated no difference of EIN regardless of formulas (4 RCTs, RR 1.04, 95% CI 0.52–2.10), Arg+RNA (1 RCT, RR 3.24, 95% CI 0.59–15.26) and Arg+RNA+ω-3-FAs (3 RCTs, RR 0.78, 95% CI 0.36–1.71) in reducing NICs compared to SEN (see Supplementary Figure 3B).

We also performed network meta-analysis to investigate comparative efficacies of EIN formulas comparing to each other or SEN in controlling NICs, but no significant differences were detected in all comparisons (see Figure 3B).

The SUCRA of Arg+RNA, Arg+RNA+ω-3-FAs and SEN was 6.36%, 83.62% and 60.02% respectively in managing NICs (see Supplementary Figure 4B). The rankings of all nutrition support regimes were delineated in Figure 4.

### Length of hospital stay

Of all 11 eligible RCTs, 9 [3, 6, 10–13, 15, 16, 18] investigated the postoperative LOS directly comparing EIN formulas with SEN. Compared to SEN, EIN regardless of formulas (9 RCTs, MD -0.42, 95% CI -0.74—0.10), Arg+RNA+ω-3-FAs (5 RCTs, MD -0.58, 95% CI -0.98—0.17) and Arg+Gln+ω-3-FAs (1 RCT, MD -0.69, 95% CI -1.22—0.17), but not Arg+RNA (1 RCT, MD 0.08, 95% CI -0.18–0.34) or Arg+Gln (2 RCTs, MD -0.20, 95% CI -0.85–0.46), significantly shortened the LOS (see Supplementary Figure 3C).

Network meta-analysis was performed to analyze the comparative efficacies of EIN formulas comparing to each other or SEN. Result from network meta-analysis indicated potential of Arg+RNA+ω-3-FAs in shortening LOS compared to SEN. However, remaining comparisons were not identified to be significantly different (see Figure 3C).

The SUCRA of Arg+RNA, Arg+RNA+ω-3-FAs, Arg+Gln, Arg+Gln+ω-3-FAs, and SEN was 18.46%, 73.86%, 48.12%, 87.01%, and 22.55% respectively (see Supplementary Figure 4C). The rankings of nutrition support regimes were delineated in Figure 4.

### Additional analysis

It's remarkable that regional databases were selected to identify potentially eligible citations may cause selection bias, so we performed sensitive analyses through excluding eligible studies retrieved from CNKI. For direct comparisons, all results from meta-analyses are with similar effect size and direction after performed sensitive analyses (see Supplementary Table 2). In network meta-analyses, the results of Arg+RNA compared to Arg+RNA+ω-3-FAs and Arg+RNA+ω-3-FAs compared to Arg Gln in reducing infectious complications and Arg+RNA+ω-3-FAs compared to SEN in shortening length of hospitalization were changed to be not significant (see Supplementary Figures 5A–7A).

In the present study, all eligible studies completed in Europe and Asia, and thus we performed subgroup analysis based on research regions (Europe and Asia). The results revealed that EIN and Arg+RNA+ω-3-FAs significantly reduced the infectious complications in Europe and Asia respectively as compared with SEN (see Supplementary Table 2). The results of noninfectious complications and length of hospitalization were not changed (see Supplementary Table 2). In the network meta-analyses, the results of Arg+RNA+ω-3-FAs as compared with Arg+Gln based on these studies performed in Asia and Arg+RNA+ω-3-FAs as compared with SEN based on these studies performed in Europe in reducing infectious complications were changed to be no significant different respectively (see Supplementary Figure 5B and 5C). The results of Arg+RNA+ω-3-FAs as compared with SEN based on these studies performed in Europe and Asia were all changed to be no significant different (see Supplementary Figure 7B and 7C). The remaining result was not significantly changed (see Supplementary Figure 6B).

After apprising the risk of bias, no eligible studies was valued as high risk of bias in any domains. And thus, we performed subgroup analyses based on the number of unclear risk of bias. In the direct meta-analyses, the pooled results of EIN as compared with SEN in reducing infectious complications were no significant when the article(s) with one, two or three domain(s) of unclear risk of bias were summarized respectively, however the result from the studies with four domains of unclear risk of bias was also significant different between these two regimes (see Supplementary Table 2). The result of EIN as compared with SEN in shortening length of hospitalization was no significant different when we pooled the data from the studies with one domain of unclear risk of bias (see Supplementary Table 2). In the network meta-analyses, the results of Arg+RNA+ω-3-FAs as compared with Arg+Gln in reducing infectious complications based on these studies with four domains of unclear risk of bias was changed to be no significant different (see Supplementary Figure 5E). The remaining results were not significantly changed (see Supplementary Figures 5D, 7D, and 7E)

Because most researches included in our study didn't report any detailed information about tumor stage, and thus we did not perform the subgroup analysis according to two these criteria.

### Investigation of inconsistency

The assessment of consistency was not performed due to no closed-loops were constructed in our study. However, in the analysis of ICs, 11 eligible RCTs were included, and thus, we drew comparison-adjusted funnel to test small-study effect. The funnel plot indicated asymmetrical graph (see Supplementary Figure 8), and thus suggested that the pooled results may be negatively impacted by small study effects.

### Rating of evidence quality

We investigated 3 outcomes of interest in this study including ICs, NICs and LOS. The qualities of evidences from direct and network meta-analysis were documented in Table 2. The quality of direct evidence ranged from very low to moderate. The potential of EIN regardless of formulas, Arg+RNA+ω-3-FAs and Arg+Gln+ω-3-FAs in decreasing ICs compared to SEN were supported by moderate, moderate and low quality evidence respectively. For shortening of LOS, low, low and moderate quality evidence supported the potential of EIN regardless of formulas, Arg+RNA+ω-3-FAs and Arg+Gln+ω-3-FAs. For overall network meta-analysis, moderate quality evidence supported that Arg+RNA+ω-3-FAs and Arg+Gln+ω-3-FAs were superior to SEN and Arg+RNA+ω-3-FAs was superior to Arg+Gln in reducing ICs; moreover, the evidence of supporting the use of Arg+RNA+ω-3-FAs rather than Arg+RNA was supported by very low quality. Low quality of evidence supported the superiority of Arg+RNA+ω-3-FAs in shortening LOS compared to SEN.

**Table 2:** The quality of evidence of all comparisons

Comparisons	Direct estimate		Indirect estimate		Network meta-analysis	
	RR (95% CI)	Quality of evidence	RR (95% CI)	Quality of evidence	OR (95% Crl)	Quality of evidence
**Infectious complications**						
**IEN vs. SEN**	**0.56 (0.36, 0.86)**	Moderate^1^	–	–	–	–
**A vs. SEN**	1.05 (0.80, 1.39)	Very low^1,2,4^	Not estimable^3^	Not estimable^3^	1.18 (0.37, 2.89)	Very low
**A vs. B**	–	–	**5.06 (1.26, 14.93)**	Very low^4^	**5.06 (1.26, 14.93)**	Very low
**A vs. C**	–	–	1.62 (0.29, 5.19)	Low^5^	1.62 (0.29, 5.19)	Low
**A vs. D**	–	–	15.39 (0.99, 77.21)	Very low^4^	15.39 (0.99, 77.21)	Very low^4^
**B vs. SEN**	**0.37 (0.22, 0.63)**	Moderate^1^	Not estimable^3^	Not estimable^3^	**0.27 (0.12, 0.49)**	Moderate
**B vs. C**	–	–	**0.36 (0.09, 0.97)**	Moderate	**0.36 (0.09, 0.97)**	Moderate
**B vs. D**	–	–	3.38 (0.26, 16.43)	Low^5^	3.38 (0.26, 16.43)	Low^5^
**C vs. SEN**	0.89 (0.353, 1.49)	Moderate^1^	Not estimable^3^	Not estimable^3^	0.95 (0.31, 2.26)	Moderate
**C vs. D**	–	–	12.14 (0.74, 62.18)	Very low^4^	12.14 (0.74, 62.18)	Very low^4^
**D vs. SEN**	**0.22 (0.05, 0.94)**	Low^1,2^	Not estimable^3^	Not estimable^3^	**0.22 (0.02, 0.84)**	Moderate^7^
**Non-infectious complications**						
**IEN vs. SEN**	1.04 (0.52, 2.10)	Low^1,5^	–	–	–	–
**A vs. SEN**	3.24 (0.69, 15.26)	Very low^1,2,4^	Not estimable^3^	Not estimable^3^	8.15 (0.64, 40.43)	Very low^1,2,4^
**B vs. SEN**	0.78 (0.36, 1.71)	Moderate^1^	Not estimable^3^	Not estimable^3^	0.84 (0.25, 2.10)	Moderate
**Length of hospitalization**						
**IEN vs. SEN**	**−0.42 (−0.74, −0.10)**	Low^1,6^	–	–	–	–
**A vs. SEN**	0.08 (−0.18, 0.34)	Very low^1,2,4^	Not estimable^3^	Not estimable^3^	0.08 (−0.76, 0.92)	Very low
**A vs. B**	–	–	0.68 (−0.28, 1.64)	Low	0.68 (−0.28, 1.64)	Low
**A vs. C**	–	–	0.28 (−0.79, 1.37)	Low	0.28 (−0.79, 1.37)	Low
**A vs. D**	–	–	0.76 (−0.49, 2.01)	Moderate	0.76 (−0.49, 2.01)	Moderate
**B vs. SEN**	**−0.42 (−0.75, −0.07)**	Low^1,6^	Not estimable^3^	Not estimable^3^	**−0.63 (−1.07, −0.13)**	Low
**B vs. C**	–	–	−0.40 (−1.22, 0.42)	Low	−0.40 (−1.22, 0.42)	Low
**B vs. D**	–	–	0.08 (−0.96, 1.12)	Moderate	0.08 (−0.96, 1.12)	Moderate
**C vs. SEN**	−0.20 (−0.85, 0.46)	Low^1,6^	Not estimable^3^	Not estimable^3^	−0.20 (−0.88, 0.48)	Low
**C vs. D**	–	–	0.48 (−0.66, 1.62)	Moderate	0.48 (−0.66, 1.62)	Moderate
**D vs. SEN**	**−0.69 (−1.22, −1.07)**	Moderate^1,2^	Not estimable^3^	Not estimable^3^	−0.68 (−1.61, 0.25)	oderate

^1^rated down for limitation, ^2^rated down for potential publication bias, ^3^cannot be estimated, ^4^severe imprecision, ^5^imprecision, ^6^inconsistency, ^7^greater precision, A = Arg+RNA, B = Arg+RNA+ω-3-FAs, C = Arg+Gln, D = Arg+Gln+ω-3-Fas, Arg = arginine, RNA = ribonucleic acid, ω-3-Fas = omega-3-fatty-acids, Gl*n =* glutamine, SEN = standard enteral nutrition, RR = risk ratio, OR = odds ratio, CI = confidence interval, Crl = credible interval.

## DISCUSSION

Although the incidence has decreased substantially over the past few decades, GC is still one of the most common malignant cancers [1]. Appropriate nutrition support enriched with immune nitrite plays predominant role in promoting recovery of GC patients underwent gastrectomy [4]. However, which EIN formulas should be optimally adopted to support this target population is still up for debate. To determine the best nutrition support regime for GC patients underwent gastrectomy and then facilitate evidence-informed decision-making in clinical practice, we performed the present network meta-analysis.

### Summary of main results

In this systematic review and network meta-analysis, we included 11 eligible RCTs enrolling 840 patients. After completed all analyses, we obtained several important findings: *(a)* direct evidences supporting EIN regardless of formulas significantly decreased ICs and LOS compared to SEN, with moderate and low quality respectively; *(b)* the evidences from direct and network meta-analysis indicated the potential of Arg+RNA+ω-3-FAs and Arg+Gln+ω-3-FAs in decreasing ICs and shortening LOS compared to SEN, with moderate and low quality respectively; *(c)* direct evidence with low quality indicated that Arg+Gln+ω-3-FAs significantly decreased ICs and this result was supported by moderate quality evidence from network meta-analysis; *(d)* direct evidence with moderate quality suggested efficacy of Arg+Gln+ω-3-FAs in shortening LOS, but this finding was not supported by the moderate quality evidence based on network meta-analysis; *(e)* the evidences from network meta-analysis suggested that Arg+RNA+ω-3-FAs was superior to Arg+RNA and Arg+Gln in reducing ICs, with very low and moderate quality respectively; *(f)* for reducing ICs, the ranking of all regimes was Arg+Gln+ω-3-FAs, Arg+RNA+ω-3-FAs, Arg+Gln, SEN, and Arg+RNA; *(g)* for reducing NICs, the ranking of all regimes was Arg+RNA+ω-3-FAs, SEN, and Arg+RNA; *(h)* for shortening LOS, the ranking of all regimes was Arg+Gln+ω-3-FAs, Arg+RNA+ω-3-FAs, Arg+Gln, SEN, and Arg+RNA.

### Strengths and weaknesses

Our systematic review and network meta-analysis has several strengths. Firstly, we designed comprehensive and sensitive search algorithms to capture any potential records and thus minimized the information bias. Secondly, our study not only analyzed direct evidence, but combined the evidences from direct and indirect comparisons, and thus more accurate estimates were generated. Thirdly, we ranked all nutrition regimes in terms of each outcome and rated the level of evidence, which facilitates evidence-informed decision-making. Fourthly, we just included RCTs stating the word of random in analysis, guaranteeing the reliability of pooled results.

Some limitations existed in our study also needs further discussion. Firstly, most of original RCTs included did not report the nutrition status of patients and thus subgroup analysis could not be performed, which impaired the reliability of our findings. Secondly, although subgroup analysis was designed to examine the impact of different nutrition regimes on patients with different tumor stages and nutrition status, however, details on these were not available in eligible studies. And thus, it is still unclear which patients can be benefit from the given these nutrition regimes due to relatively high heterogonous. Moreover, the power of our results may be impaired by this weakness. Thirdly, we did not perform funnel for comparisons with less than 10 RCTs [4], and thus our results may be impaired by publication bias. Fourthly, the comparison-adjusted funnel indicated asymmetric, suggesting small study effect may reduce the robustness [19]. Fifthly, the time of measuring outcomes were varying from one to another. Sixthly, some estimates in our study generated from individual RCT with small number of patients. Seventhly, regional databases were partly included to capture potential records may cause selection bias, and thus we excluded eligible studies retrieved from CNKI and found that the results of Arg + RNA compared to Arg + RNA +ω-3-FAs and Arg + RNA +ω-3-FAs compared to Arg + Gln in reducing infectious complications and Arg + RNA +ω-3-FAs compared to SEN in shortening length of hospitalization were also changed to be not significant. This is an indication that our results may be impaired by selection bias. Moreover, the powers of some summarized results were impaired by research region and the degree of risk of bias, and thus we recommend the practitioners cautiously consider our partial findings.

### Agreements and disagreements in the current literature

The present systematic review and network meta-analysis firstly investigated the comparative efficacies of various EIN formulas in supporting GC patients underwent gastrectomy. Of previous RCTs, most were performed to directly compare EIN regime with SEN and remaining studies investigated the comparative effectiveness of various routes of administration. Consequently, it is not possible to determine the efficacies of different EIN formulas in treating a certain target population [3].

Nevertheless, some investigators comprehensively evaluated the comparative efficacies of EIN in treating several types of patients compared to SEN regimes by using meta-analysis technique. Of these studies, three previous pairwise meta-analyses were performed to investigate the comparative efficacies of EIN regardless of formulas compared to SEN for the treatment of gastrointestinal malignant cancers, and indicated that EIN regimes reduced postoperative ICs [20, 21], NICs [20] and LOS [20–22]. Our study indicated EIN was effective in reducing ICs and LOS, which are consistent with the findings from previous meta-analyses enrolled gastrointestinal patients [20–22]. Moreover, two [4, 23] pairwise meta-analyses enrolled the GC patients underwent surgical resection to be as the target population, and only one [4] suggested EIN did not improve the clinical outcomes including surgical site infections (SSIs), other infectious complications (OICs) and LOS, which were contrary to our findings. Must be noted is that previous meta-analysis [4] divided the ICs into SSIs and OICs and separately analyzed these two indices. However, in the present study, the ICs were regarded as the individual outcome. So, these different analytic units may cause generation of difference results. Moreover, for shortening LOS, only 2 eligible RCTs with 291 patients were included, whereas 9 eligible RCTs enrolling 730 patients were incorporated in the present study. Pooled results generated from small numbers and small sample sizes are more vulnerable to errors. Consequently, the conclusion of EIN can shorten LOS may be more reliable. In addition, the present network meta-analysis firstly makes hierarchies of different EIN formulas including Arg+RNA, Arg+RNA+ω-3-FAs, Arg+Gln and Arg+Gln+ω-3-FAs which were not reported in previous studies.

## MATERIALS AND METHODS

We performed and reported this systematic review and network meta-analysis in accordance with the Cochrane Handbook for Systematic Reviews of Interventions [24] and preferred reporting items for systematic review and meta-analysis (PRISMA) [25] respectively. Ethical approval and patients written inform consent was not required due to the data in our study from published trials.

### Selection criteria

In present systematic review and network meta-analysis, we included the RCTs meeting following eligibility criteria: *(a) Patients*: all adults with histologically diagnosed GC who were scheduled for gastrectomy; *(b) Intervention*: all EIN formulas, regardless of administration time; *(c) Comparison*: other active EIN formulas or SEN; *(d) Outcomes*: postoperative infectious complications (ICs), postoperative non-infectious complications (NICs) and LOS.

We excluded the references meeting following one of the items: *(a)* patients with unresectable neoplasm, administration of corticosteroids or immunosuppressive agents, previous abdominal radiotherapy, active preoperative infection, underlying cardiovascular pathology, and renal or hepatic function impairment were defined as target population; *(b)* essential information cannot be extracted; *(c)* duplication with poor methodology and insufficient data; *(d)* nonoriginal research, such as review, letter and specialist comments.

### Identification of citations

We designed all sensitive search strategies by using Boolean logic operator at the basis of medical subject heading and free text. And then, these strategies were used to capture potential records that compared EIN formulas with each other or SEN in PubMed, Cochrane Central Register of Controlled Trials (CENTRAL), EMBASE, and China National Knowledge Infrastructure (CNKI), through January 2016. We also manually checked the reference lists of all eligible studies and retrieved electronically Clinicaltrial.gov, and American Society of Clinical Oncology (ASCO) to include any eligible trial. The articles published in English or Chinese language were incorporated in our systematic review and network meta-analysis. Those search strategies that were used to identify articles in English language were sorted in Supplementary Table 1.

### Data extraction

Two reviewers used the pre-designed data extraction form [19] to abstract the basic information and essential continuous and dichotomous data for specific outcome from eligible study, such as first author, publication year, age of participants, sample size, nutrition status, intervention regimes, and outcomes of interest. We contacted the corresponding author to acquire the sufficient data. Consensus principle was used as the method to resolve divergences between reviewers.

### Quality assessment of individual study

Two reviewers cautiously appraised the risk of bias of each eligible individual study by using the Cochrane risk of bias assessment tool [24, 26]. Seven domains including randomization sequence generation, allocation concealment, blinding of participants and study personnel, blinding of outcome assessors, incomplete outcome data, selective reporting and other bias were assessed accordingly, and then a study will be rated to be ‘*high risk of bias*’, ‘*unclear risk of bias*’ or ‘*low risk of bias*’ according to the match level between actual information in eligible study and evaluation criteria [24].

### Quality of evidence

We used the grading of recommendations assessment, development and evaluation (GRADE) method to rate the quality of evidence [27]. In this method, the quality of direct evidence was firstly rated to be high and five factors, which includes limitation (that is risk of bias), imprecision which can be evaluated using confidence intervals, indirectness, inconsistency which can be evaluated using heterogeneity, and publication bias which can be tested using funnel plot, can reduce the level to moderate, low and very low [27, 28]. The quality of indirect evidence was rated to be consistent with the lowest level of treatments which contribute as first-order loops to the indirect evidence [27, 28] and the imprecision and intransitivity can further reduce the level [27, 28]. If the assumption of agreement of estimates between direct and indirect comparisons was established, the quality of evidence from network meta-analysis would be rated by using the higher of their level [27, 28].

### Statistical analysis

We firstly performed pairwise meta-analysis based on random effect model, which incorporates within- and between-studies heterogeneity, to estimate the summarized risk ratio (RR), mean difference (MD) and 95% confidence intervals (CIs) [29]. We adopted Chi^2^ method to test the heterogeneity [30] and used I^2^ statistic to estimate the proportion of the overall variation that is attributable to between-study heterogeneity [31]. The value of I^2^ statistic was larger than 50% indicating substantial heterogeneity [31]. We also drew the funnel plot to identify publication bias when the number of studies analyzed was more than 10 [32].

After completed the pairwise meta-analysis, we performed random-effects network meta-analysis using the Markov chain Monte Carlo (MCMC) simulation from the posterior distribution to calculate the estimates of relative effects and all model parameters following methods described by Chaimani and colleagues [33]. We used starting value which automatically generated from software to fit the model [34]. To gain convergence, we performed each MCMC chain with 70000 iterations and 30000 burn-in. We have drawn the comparison-adjusted funnel plot to assess the small-study effects when the number of studies included in one pair of comparison was more than 10 [35]. We calculated the surface under the cumulative ranking curve (SUCRA) to rank all EIN formulas and the higher SUCRA value was correspond to better results for respective treatment [36]. We did not assess the consistency between direct and indirect estimates due to no loop was constructed in our study [33]. We will perform sensitive analysis through excluding eligible studies retrieved from regional database such as CNKI to examine selection bias. Subgroup analysis will be also designed according to the nutritional status and tumor stage when details can be extracted from eligible studies because tumor stage is a significant factor causing and deteriorating malnutrition. Moreover, we will also perform subgroup analysis according to the research regions and degree of risk of bias of all eligible studies.

All analyses were conducted by using the RevMan 5.3 (Copenhagen: The Nordic Cochrane Centre, The Cochrane Collaboration, 2013), Stata 12 (StataCorp, Texas) and WinBUGS 1.4 (imperial College School of Medicine at St Mary's, London).

## CONCLUSIONS

In summary, we identified several important conclusions with significant implications for clinical practice and further research by performing this systematic review and network meta-analysis. Firstly, EIN is an effective nutrition support regime of promoting recovery of GC patients underwent gastrectomy. Secondly, Arg+Gln+ω-3-FAs and Arg+RNA+ω-3-FAs are the optimal regimes of reducing ICs and LOS; it must be noted is that, however, the use of Arg+RNA in controlling ICs, NICs and LOS are not preferentially recommended compared to SEN. Moreover, most findings in our study generated from small numbers with small sample sizes, and most importantly, the administration time of nutrition support, time of measuring outcomes and nutrition status of patients are different among eligible studies, so these findings in our study should be cautiously interpreted and on the other hand, more multicenter RCTs with larger scale, targeted patients with comparative characteristics and good design are urgently required. Moreover, because regional database was included in our manuscript and sensitive analysis found possible selection bias, and thus a systematic review with more comprehensive literature retrieval and international original studies should be designed in order to avoid selection bias. We also did not capture RCTs directly comparing EIN formulas with each other, and thus larger studies with good design are warranted.

## SUPPLEMENTARY MATERIALS FIGURES AND TABLES





## Abbreviations

EIN: enteral immunonutrition
SEN: standard enteral nutrition
GC: gastric cancer
ICs: infectious complications
NICs: noninfectious complications
LOS: length of hospital stay
SUCRA: surface under the cumulative ranking curve
GRADE: Grading of Recommendations Assessment, Development and Evaluation
CI: confidence interval
RR: risk ratio
MD: mean difference
OR: odds ratio
CrI: credibility interval
SMD: standard mean difference
Arg: arginine
Gln: glutamine
ω-3-Fas: omega-3 fatty acids
RNA: ribonucleic acid
RCTs: randomized controlled trials
PRISMA: Preferred Reporting Items for Systematic Reviews and Meta-Analyses
