# Supplementary material for: Systematic review with network meta-analysis: comparative efficacy of different enteral immunonutrition formulas in patients underwent gastrectomy
Source: Oncotarget. 2017 Feb 21;8(14):23376–88. doi: 10.18632/oncotarget.15580 (PMC5410311; doi:10.18632/oncotarget.15580)
Supplement: Supplementary file 1 [file oncotarget-08-23376-s001.pdf]

Systematic review with network meta-analysis: comparative efficacy of different enteral immunonutrition formulas in patients underwent gastrectomy

Supplementary Materials

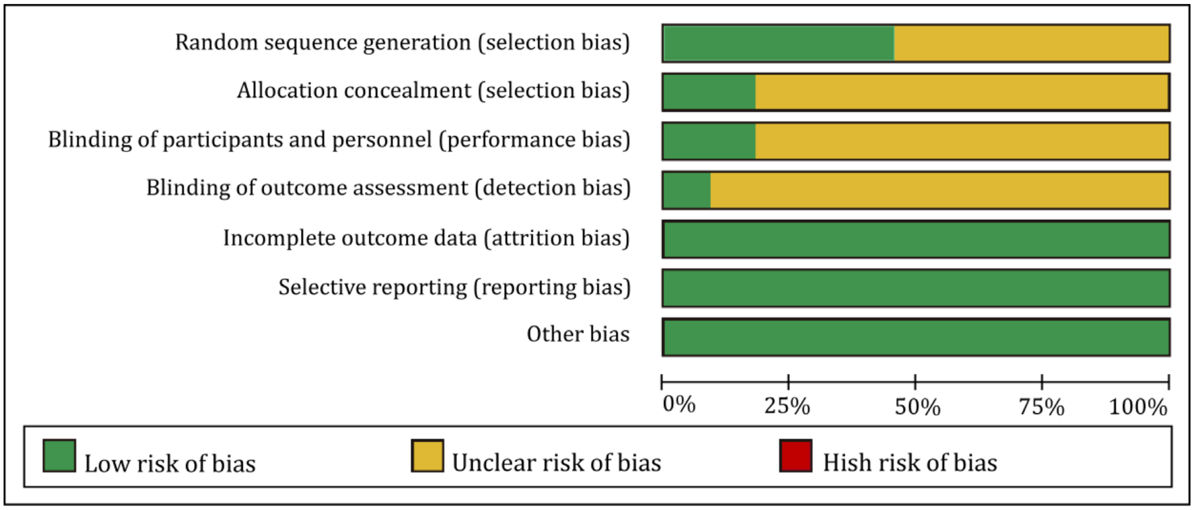

Supplementary Figure 1: The cumulative percentages graph for each risk of bias domain. The yellow, green and red represent "unclear risk of bias", "low risk of bias" and "high risk of bias" respectively. The wide of a color indicated percentages of risk of bias.

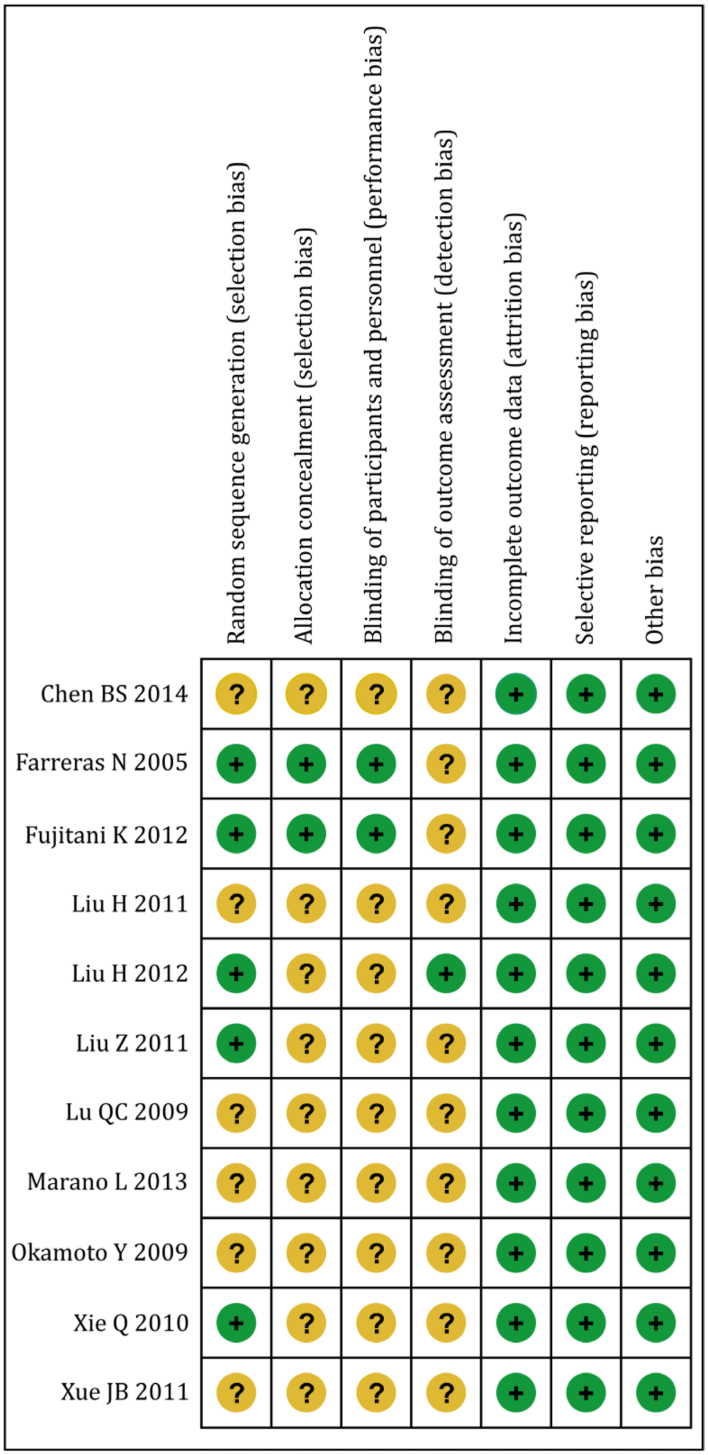

Supplementary Figure 2: Risk of bias summary for individual randomized controlled trials. The yellow (question mark), green (plus sign) and red (minus sign) represent “unclear risk of bias”, “low risk of bias” and “high risk of bias” respectively.

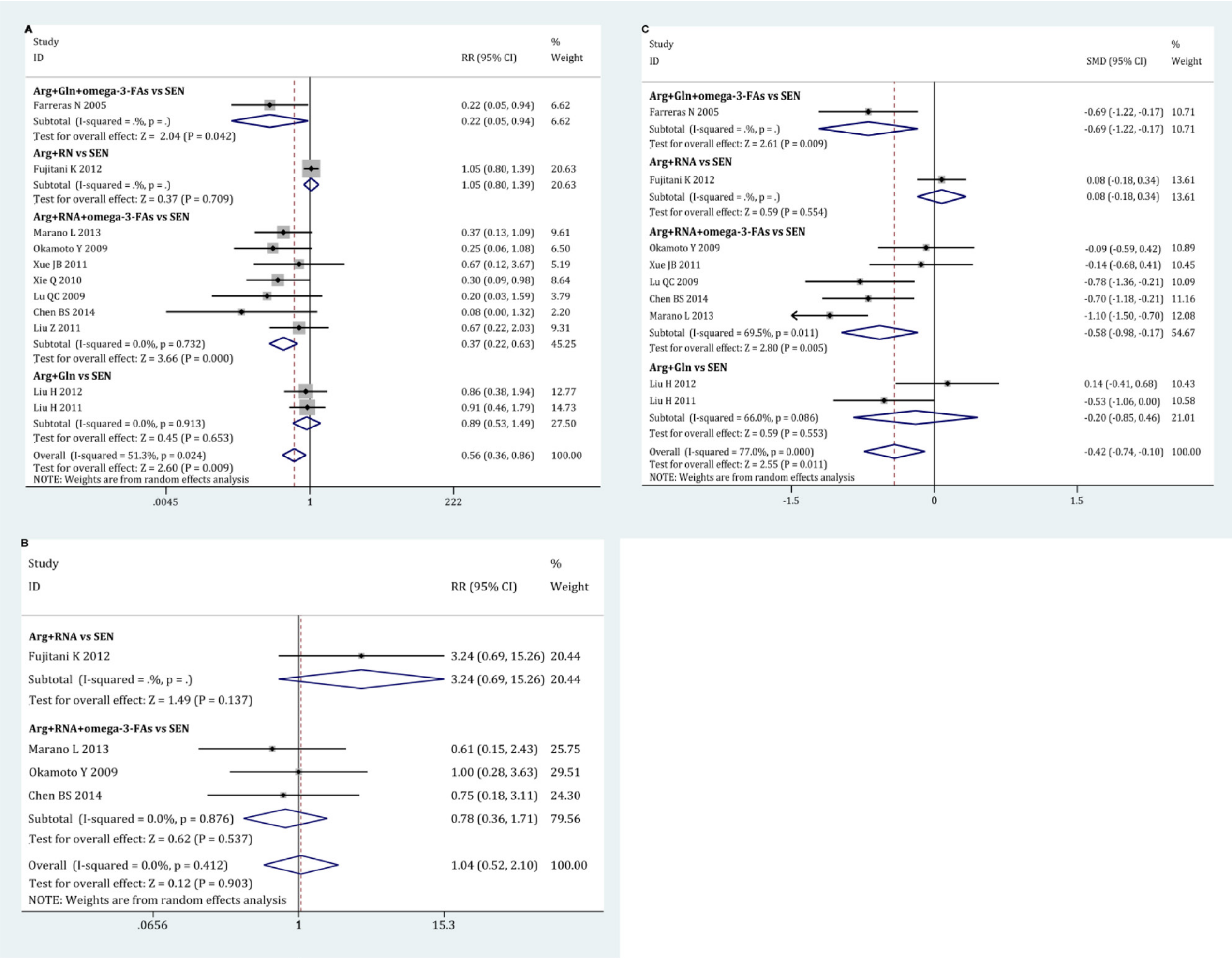

Supplementary Figure 3: Forest plot for the outcomes of infectious complications (**A**), noninfectious complications (**B**) and the length of hospital stay (**C**) with enteral immunonutrition (EIN) formulas versus standard enteral nutrition (SEN). The summary effect estimate (risk ratio, RR; standardized mean difference, SMD) for individual randomized controlled trials (RCTs) are indicated by grey rectangles (the size of the rectangle is proportional to the study weight), with the black horizontal lines representing 95% confidence intervals (CIs). The overall summary effect estimate (risk ratio or standardized mean difference) and 95% confidence interval are indicated by the blue diamond below.

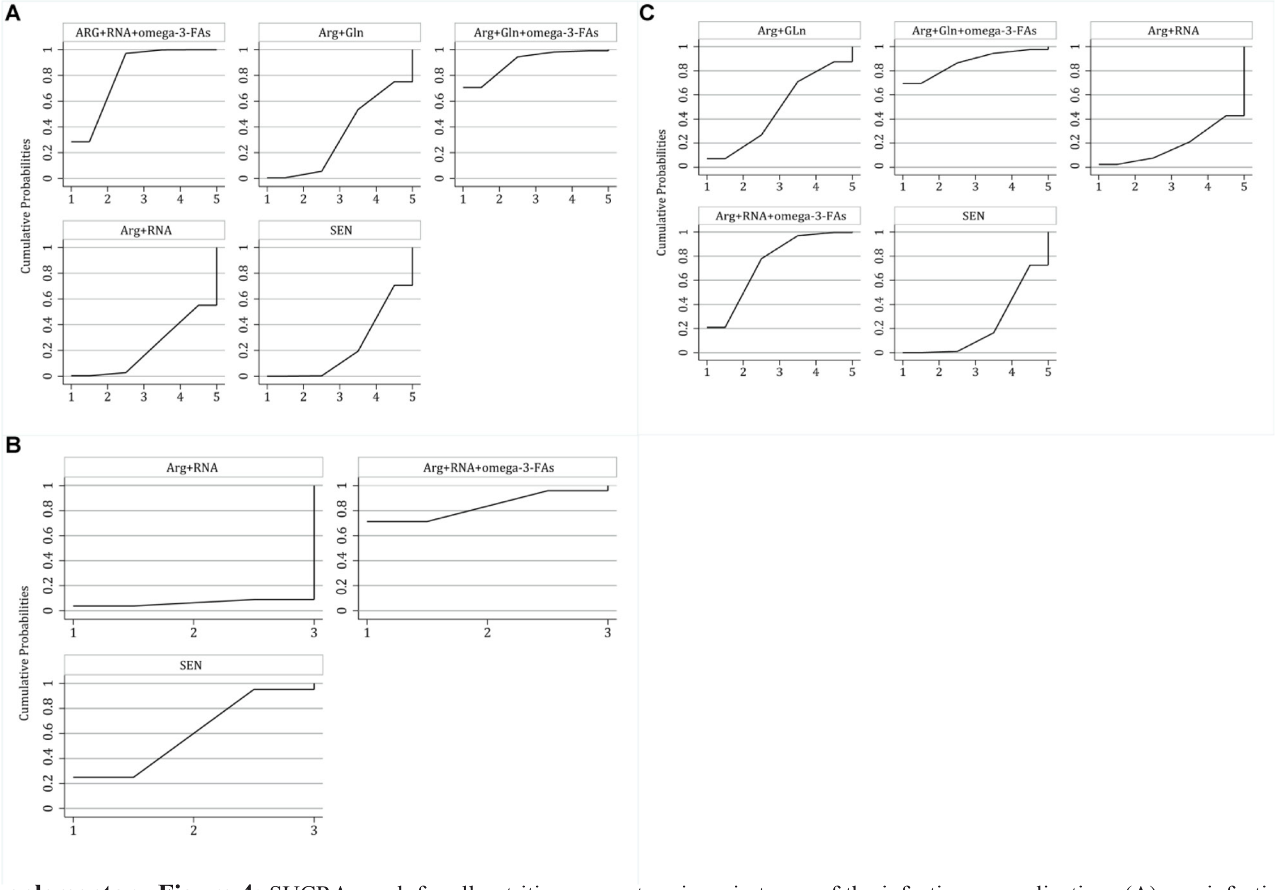

Supplementary Figure 4: SUCRA graph for all nutrition support regimes in terms of the infectious complications (**A**), noninfectious complications (**B**) and the length of hospital stay (**C**). The number presented in vertical line indicated cumulative probabilities of becoming best efficacious option.

Arg+RNA	NA	NA	NA	1.05 (0.80, 1.39)
4.42 (0.69, 15.44)	Arg+RNA+ω-3-FAs	NA	NA	**0.40 (0.20, 0.80)**
2.18 (0.19, 9.23)	0.65 (0.06, 2.64)	Arg+Gln	NA	0.89 (0.353, 1.49)
16.88 (0.80, 90.17)	4.836 (0.27, 25.2)	14.69 (0.47, 81.8)	Arg+Gln+ω-3-FAs	**0.22 (0.05, 0.94)**
1.23 (0.31, 3.43)	**0.37 (0.11, 0.85)**	1.08 (0.16, 3.77)	**0.23 (0.02, 0.92)**	SEN

Treatments   Direct comparisons   Indirect comparisons

Supplementary Figure 5A: Sensitive analysis on infectious complications of different treatments through excluding studies retrieved from regional databases.

Arg+RNA	NA	NA	1.05 (0.80, 1.39)
5.69 (1.20, 17.85)	Arg+RNA+ω-3-FAs	NA	**0.32 (0.18, 0.59)**
1.67 (0.27, 5.49)	0.35 (0.07, 1.00)	Arg+Gln	0.89 (0.353, 1.49)
1.21 (0.35, 3.13)	**0.25 (0.10, 0.51)**	0.96 (0.30, 2.36)	SEN

Treatments   Direct comparisons   Indirect comparisons

Supplementary Figure 5B: Sensitive analysis on infectious complications of different treatments based on studies performed in Asia.

Arg+RNA+ω-3-FAs	NA	0.37 (0.13, 1.09)
6.23 (0.16, 35.14)	Arg+Gln+ω-3-FAs	**0.22 (0.05, 0.94)**
0.42 (0.05, 1.53)	**0.23 (0.01, 0.97)**	SEN

Treatments   Direct comparisons   Indirect comparisons

Supplementary Figure 5C: Sensitive analysis on infectious complications of different treatments based on studies performed in Europe.

Arg+RNA	NA	1.05 (0.80, 1.39)
17.93 (0.75, 95.56)	Arg+Gln+ω-3-FAs	**0.22 (0.05, 0.94)**
1.27 (0.28, 3.78)	**0.23 (0.01, 0.94)**	SEN

Supplementary Figure 5D: Sensitive analysis on infectious complications of different treatments based on these studies which were valued unclear risk of bias in 1 domain.

Arg+RNA+ω-3-FAs	NA	**0.29 (0.14, 0.57)**
0.637 (0.04, 1.42)	Arg+Gln	0.91 (0.46, 1.79)
**0.23 (0.08, 0.51)**	1.15 (0.19, 3.91)	SEN

Treatments Direct comparisons Indirect comparisons

Supplementary Figure 5E: Sensitive analysis on infectious complications of different treatments based on these studies which were valued unclear risk of bias in 4 domains. The upper right area presented the effect sizes of direct comparisons and the bottom left shown the direct comparisons. For direct comparison, it favors the row-defining treatment if ORs lower than 1. In contrast, for indirect comparison, the result favors the column-defining treatment if odds ratios (ORs) lower than 1. A number with bold font indicated a significant difference between two treatments. SEN: standard enteral nutrition.

Arg+RNA	NA	3.24 (0.69, 15.26)
13.72 (0.53, 77.27)	Arg+RNA+ω-3-FAs	0.79 (0.31, 2.00)
8.23 (0.60, 40.64)	0.93 (0.20, 2.75)	SEN

Treatments Direct comparisons Indirect comparisons

Supplementary Figure 6A: Sensitive analysis on non-infectious complications of different treatments through excluding studies retrieved from regional databases.

Arg+RNA	NA	3.24 (0.69, 15.26)
12.33 (0.47, 68.72)	Arg+RNA+ω-3-FAs	0.88 (0.34, 2.27)
8.27 (0.63, 41.58)	1.07 (0.22, 3.21)	SEN

Treatments Direct comparisons Indirect comparisons

Supplementary Figure 6B: Sensitive analysis on non-infectious complications of different treatments based on studies performed in Asia. The upper right area presented the effect sizes of direct comparisons and the bottom left shown the direct comparisons. For direct comparison, it favors the row-defining treatment if ORs lower than 1. In contrast, for indirect comparison, the result favors the column-defining treatment if odds ratios (ORs) lower than 1. A number with bold font indicated a significant difference between two treatments. SEN: standard enteral nutrition.

Arg+RNA	NA	NA	NA	0.08 (-0.18, 0.34)
0.39 (-0.81, 1.60)	Arg+RNA+ω-3-FAs	NA	NA	**-0.42 (-0.75, -0.07)**
-0.06 (-1.50, 1.41)	-0.45 (-1.74, 0.84)	Arg+Gln	NA	0.14 (-0.41, 0.68)
0.77 (-0.66, 2.22)	0.38 (-0.89, 1.65)	0.83 (-0.67, 2.31)	Arg+Gln+ω-3-FAs	**-0.71 (-1.02, -0.39)**
0.08 (-0.90, 1.07)	-0.31 (-1.04, 0.41)	0.14 (-0.93, 1.19)	-0.69 (-1.75, 0.36)	SEN

Treatments Direct comparisons Indirect comparisons

Supplementary Figure 7A: Sensitive analysis on lengths of hospitalization of different treatments through excluding studies retrieved from regional databases.

Arg+RNA	NA	NA	0.08 (-0.18, 0.34)
0.50 (-0.55, 1.56)	Arg+RNA+ω-3-FAs	NA	**-0.42 (-0.68, -0.16)**
0.29 (-0.89, 1.45)	-0.22 (-1.08, 0.66)	Arg+Gln	-0.20 (-0.85, 0.46)
0.08 (-0.85, 1.01)	-0.42 (-0.93, 0.08)	-0.20 (-0.92, 0.51)	SEN

Treatments Direct comparisons Indirect comparisons

Supplementary Figure 7B: Sensitive analysis on lengths of hospitalization of different treatments based on studies performed in Asia.

Arg+RNA+ω-3-FAs	NA	**-1.10 (-1.50, -0.70)**
-0.40 (-2.14, 1.35)	Arg+Gln+ω-3-FAs	**-0.69 (-1.22, -0.17)**
-1.09 (-2.31, 0.11)	-0.69 (-1.95, 0.57)	SEN

Treatments   Direct comparisons   Indirect comparisons

Supplementary Figure 7C: Sensitive analysis on lengths of hospitalization of different treatments based on studies performed in Europe.

Arg+RNA	NA	0.08 (-0.18, 0.34)
0.77 (-0.97, 2.49)	Arg+Gln+ω-3-FAs	**-0.69 (-1.22, -0.17)**
0.07 (-1.12, 1.27)	-0.69 (-1.94, 0.57)	SEN

Treatments   Direct comparisons   Indirect comparisons

Supplementary Figure 7D: Sensitive analysis on lengths of hospitalization of different treatments based on these studies which were valued unclear risk of bias in 1 domain.

Arg+RNA+ω-3-FAs	NA	**-0.58 (-0.98, -0.17)**
-0.05 (-1.30 1.20)	Arg+Gln	-0.53 (-1.06, 0.00)
**-0.57 (-1.07, -0.06)**	-0.52 (-1.66, 0.62)	SEN

Treatments   Direct comparisons   Indirect comparisons

Supplementary Figure 7E: Sensitive analysis on lengths of hospitalization of different treatments based on these studies which were valued unclear risk of bias in 4 domains. Each number in each cell represents the effect size of the treatment in upper left area minus the treatment in bottom right area. Standard mean differences (SMDs) lower than 0 favor the column-defining treatment. The upper right area presented the effect sizes of direct comparisons and the bottom left shown the direct comparisons. A number with bold font indicated a significant difference between two treatments. SEN: standard enteral nutrition.

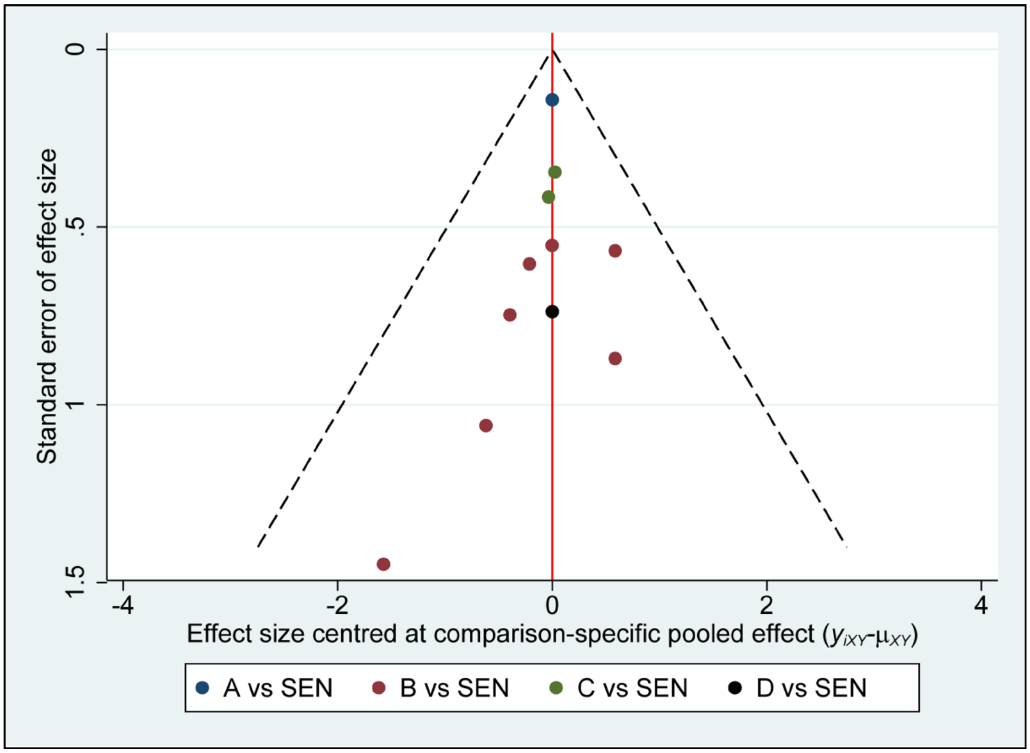

Supplementary Figure 8: Comparison-adjusted funnel for infectious complications with enteral immunonutrition versus standard enteral nutrition. The vertical axis represented the standard error (SE) of effect size and x axis indicated the difference between effect size and pooled effect. Asymmetrical funnel plot indicated small study effect. The A represented Arg+RNA, B represented Arg+RNA+ω-3-FAs, C represented Arg+Gln, and D represented Arg+Gln+omega-3-FAs. Arg = arginine, RNA = ribonucleic acid, ω-3-FAs = omega-3-fatty-acids, Gln = glutamine.

Supplementary Table 1: CENTRAL Search Algorithm. See Supplementary_Table_1

Supplementary Table 2: Sensitive analysis

Excluding studies retrieved from regional database

Comparisons	No. of included studies	Heterogeneity (%)	RR/MD (95% CI)
Infectious complications			
EIN *vs* SEN	6	56.7	0.60 (0.34, 1.05)
Arg+RNA+ω-3-FAs *vs* SEN	3	0.0	0.40 (0.20, 0.80)
Arg+Gln vs SEN	1	n.a.	0.86 (0.38, 1.94)
Non-infectious complications			
EIN *vs* SEN	3	22.8	1.18 (0.47, 2.96)
Arg+RNA+ω-3-FAs *vs* SEN	2	0.0	0.79 (0.31, 2.00)
Length of hospitalization			
EIN *vs* SEN	5	85.9	–0.25 (–0.43, –0.07)
Arg+RNA+ω-3-FAs *vs* SEN	2	89.4	–0.71 (–1.02, –0.39)

Based on studies performed in different regions

Comparisons	Region	No. of included studies	Heterogeneity (%)	RR/MD (95% CI)
Infectious complications				
EIN *vs* SEN	Europe	2	0.0	0.31 (0.13, 0.73)
	Asia	9	43.5	0.66 (0.42, 1.02)
Arg+RNA+ω-3-FAs *vs* SEN	Europe	1	n.a.	0.37 (0.13, 1.09)
	Asia	6	0.0	0.32 (0.18, 0.59)
Non-infectious complications				
EIN *vs* SEN	Europe	1	n.a.	0.61 (0.15, 2.43)
	Asia	3	4.6	1.26 (0.55, 2.90)
Arg+RNA+ω-3-FAs *vs* SEN	Europe	1	n.a.	0.61 (0.15, 2.43)
	Asia	2	0.0	0.88 (0.34, 2.27)
Length of hospitalization				
EIN *vs* SEN	Europe	2	31.2	–0.95 (–1.27, –0.63)
	Asia	7	61.3	–0.18 (–0.34, –0.01)
Arg+RNA+ω-3-FAs *vs* SEN	Europe	1	n.a.	–1.10 (–1.50, –0.70)
	Asia	4	45.1	–0.42 (–0.68, –0.16)

According to the degree of ROB

Comparisons	NUROB	No. of included studies	Heterogeneity (%)	RR/MD (95% CI)
Infectious complications				
EIN *vs* SEN	1	2	77.9	0.57 (0.12, 2.63)
	2	1	n.a.	0.86 (0.38, 1.94)
	3	2	0.0	0.46 (0.20, 1.03)
	4	6	31.6	0.45 (0.23, 0.88)
Arg+RNA+ω-3-FAs *vs* SEN	4	5	0.0	0.29 (0.14, 0.57)
	3	2	0.0	0.44 (0.20, 0.97)
Arg+Gln *vs* SEN	2	1	n.a.	0.86 (0.38, 1.94)
	4	1	n.a.	0.91 (0.46, 1.79)
Non-infectious complications				
EIN *vs* SEN	1	1	n.a.	3.24 (0.69, 15.26)
	4	3	0.0	0.78 (0.36, 1.71)
Length of hospitalization				
EIN *vs* SEN	1	2	85.2	–0.07 (–0.31, 0.16)
	2	1	n.a.	–0.53 (–1.06, 0.00)
	4	6	62.2	–0.61 (–0.81, –0.41)
Arg+Gln *vs* SEN	2	1	n.a.	0.14 (–0.41, 0.68)
	4	1	n.a.	–0.53 (–1.06, 0.00)

OR, odds ratio; MD, mean difference; CI, confidence interval; EIN, enteral immunonutrition; Arg, arginine; Gln, glutamine; RNA, ribonucleic acid; ω-3-FA, omega-3-fatty acids; ROB, risk of bias; NUROB, number of unclear risk of bias; n.a., not available
