# Supplementary material for: Systematic review with network meta-analysis: comparative efficacy of different enteral immunonutrition formulas in patients underwent gastrectomy
Source: Oncotarget. 2017 Feb 21;8(14):23376–88. doi: 10.18632/oncotarget.15580 (PMC5410311; doi:10.18632/oncotarget.15580)
Supplement: Supplementary file 2 [file oncotarget-08-23376-s002.docx]

|  |
| --- |

**CENTRAL Search Algorithm**

**ID Search**

#1 "gastric cancer":ti,ab,kw or "gastric carcinoma":ti,ab,kw or gastric neoplasm*:ti,ab,kw or stomach neoplasm*:ti,ab,kw or gastric tumor*:ti,ab,kw in Trials (Word variations have been searched)

#2 "stomach cancer":ti,ab,kw or stomach carcinoma*:ti,ab,kw or gastric tumor*:ti,ab,kw (Word variations have been searched)

#3 #1 or #2

#4 MeSH descriptor: [Stomach Neoplasms] explode all trees

#5 #3 or #4

#6 L-Arginine:ti,ab,kw or "arginine":ti,ab,kw or DL-Arginine Acetate, Monohydrate:ti,ab,kw or DL Arginine Acetate, Monohydrate:ti,ab,kw (Word variations have been searched)

#7 MeSH descriptor: [Arginine] explode all trees

#8 #6 or #7

#9 Fatty Acids, Omega 3:ti,ab,kw or "omega-3 fatty acid":ti,ab,kw or "omega 3 fatty acids":ti,ab,kw or n-3 Fatty Acid*:ti,ab,kw or n-3 PUFA:ti,ab,kw (Word variations have been searched)

#10 MeSH descriptor: [Fatty Acids, Omega-3] explode all trees

#11 #9 or #10

#12 "glutamine":ti,ab,kw or L-Glutamine:ti,ab,kw or D-Glutamine:ti,ab,kw (Word variations have been searched)

#13 MeSH descriptor: [Glutamine] explode all trees

#14 #12 or #13

#15 RNA:ti,ab,kw or RNA Gene Product*:ti,ab,kw or "ribonucleic acid":ti,ab,kw or RNA, Non-Polyadenylated:ti,ab,kw or RNA, Non Polyadenylated:ti,ab,kw (Word variations have been searched)

#16 MeSH descriptor: [RNA] explode all trees

#17 #15 or #16

#18 immunonutrition:ti,ab,kw or immunutrition:ti,ab,kw or immune nutrition:ti,ab,kw (Word variations have been searched)

#19 #8 or #11 or #14 or #17 or #18

#20 "nutritional support":ti,ab,kw or "artificial feeding":ti,ab,kw or diet supplementation:ti,ab,kw (Word variations have been searched)

#21 MeSH descriptor: [Nutritional Support] explode all trees

#22 #20 or #21

#23 "enteral nutrition*":ti,ab,kw or Enteral Feeding:ti,ab,kw or "force feeding*":ti,ab,kw or Gastric Feeding Tube:ti,ab,kw or "tube feeding*":ti,ab,kw (Word variations have been searched)

#24 Enteric Feeding*:ti,ab,kw or "diet therapy":ti,ab,kw (Word variations have been searched)

#25 #23 or #24

#26 MeSH descriptor: [Enteral Nutrition] explode all trees

#27 #25 or #26

#28 #22 or #27

#29 random*:ti,ab,kw or Clinical Trials, Phase III:ti,ab,kw or Clinical Trials, Phase IV:ti,ab,kw (Word variations have been searched)

#30 MeSH descriptor: [Randomized Controlled Trial] explode all trees

#31 MeSH descriptor: [Randomized Controlled Trials as Topic] explode all trees

#32 MeSH descriptor: [Controlled Clinical Trial] explode all trees

#33 MeSH descriptor: [Controlled Clinical Trials as Topic] explode all trees

#34 MeSH descriptor: [Clinical Trials, Phase III as Topic] explode all trees

#35 MeSH descriptor: [Clinical Trials, Phase IV as Topic] explode all trees

#36 #30 or #31 or #32 or #33 or #34 or #35

#37 #29 or #36

#38 #5 and #19 and 28 and #37

| **PubMed Search Algorithm** |
| --- |

| **Search** | **Query** |
| --- | --- |
| #45 | Search ((((("Stomach Neoplasms"[Mesh]) OR ((((((((gastric cancer*[Title/Abstract]) OR gastric carcinoma*[Title/Abstract]) OR gastric neoplasm*[Title/Abstract]) OR stomach neoplasm*[Title/Abstract]) OR gastric tumor*[Title/Abstract]) OR stomach cancer*[Title/Abstract]) OR stomach carcinoma*[Title/Abstract]) OR gastric tumor*[Title/Abstract]))) AND ((((((("Arginine"[Mesh]) OR ((((L-Arginine[Title/Abstract]) OR Arginine[Title/Abstract]) OR DL-Arginine Acetate, Monohydrate[Title/Abstract]) OR DL Arginine Acetate, Monohydrate[Title/Abstract]))) OR (("Fatty Acids, Omega-3"[Mesh]) OR (((((Fatty Acids, Omega 3[Title/Abstract]) OR Omega-3 Fatty Acids[Title/Abstract]) OR omega 3 fatty acids[Title/Abstract]) OR n-3 Fatty Acids[Title/Abstract]) OR n-3 PUFA[Title/Abstract]))) OR (("Glutamine"[Mesh]) OR (((glutamine[Title/Abstract]) OR L-Glutamine[Title/Abstract]) OR D-Glutamine[Title/Abstract]))) OR (("RNA"[Mesh]) OR (((((RNA[Title/Abstract]) OR RNA Gene Product[Title/Abstract]) OR Ribonucleic Acid[Title/Abstract]) OR RNA, Non-Polyadenylated[Title/Abstract]) OR RNA, Non Polyadenylated[Title/Abstract]))) OR (((immunonutrition[Title/Abstract]) OR immunutrition[Title/Abstract]) OR immune nutritio[Title/Abstract]))) AND (((("Nutritional Support"[Mesh]) OR (((Nutritional Support[Title/Abstract]) OR Artificial Feeding[Title/Abstract]) OR diet supplementation[Title/Abstract]))) OR (("Enteral Nutrition"[Mesh]) OR (((((((enteral nutrition[Title/Abstract]) OR Enteral Feeding*[Title/Abstract]) OR Force Feeding*[Title/Abstract]) OR Gastric Feeding Tube*[Title/Abstract]) OR Tube Feeding*[Title/Abstract]) OR Enteric Feeding[Title/Abstract]) OR Diet Therapy[Title/Abstract])))) AND (((((((("Randomized Controlled Trial" [Publication Type]) OR "Randomized Controlled Trials as Topic"[Mesh]) OR "Controlled Clinical Trial" [Publication Type]) OR "Controlled Clinical Trials as Topic"[Mesh]) OR "Clinical Trials, Phase III as Topic"[Mesh]) OR "Clinical Trials, Phase IV as Topic"[Mesh])) OR (((random*[Title/Abstract]) OR Clinical Trials, Phase III[Title/Abstract]) OR Clinical Trials, Phase IV[Title/Abstract])) |
| #44 | Search ((((((("Randomized Controlled Trial" [Publication Type]) OR "Randomized Controlled Trials as Topic"[Mesh]) OR "Controlled Clinical Trial" [Publication Type]) OR "Controlled Clinical Trials as Topic"[Mesh]) OR "Clinical Trials, Phase III as Topic"[Mesh]) OR "Clinical Trials, Phase IV as Topic"[Mesh])) OR (((random*[Title/Abstract]) OR Clinical Trials, Phase III[Title/Abstract]) OR Clinical Trials, Phase IV[Title/Abstract]) |
| #43 | Search ((random*[Title/Abstract]) OR Clinical Trials, Phase III[Title/Abstract]) OR Clinical Trials, Phase IV[Title/Abstract] |
| #42 | Search ((((("Randomized Controlled Trial" [Publication Type]) OR "Randomized Controlled Trials as Topic"[Mesh]) OR "Controlled Clinical Trial" [Publication Type]) OR "Controlled Clinical Trials as Topic"[Mesh]) OR "Clinical Trials, Phase III as Topic"[Mesh]) OR "Clinical Trials, Phase IV as Topic"[Mesh] |
| #37 | Search ((("Nutritional Support"[Mesh]) OR (((Nutritional Support[Title/Abstract]) OR Artificial Feeding[Title/Abstract]) OR diet supplementation[Title/Abstract]))) OR (("Enteral Nutrition"[Mesh]) OR (((((((enteral nutrition[Title/Abstract]) OR Enteral Feeding*[Title/Abstract]) OR Force Feeding*[Title/Abstract]) OR Gastric Feeding Tube*[Title/Abstract]) OR Tube Feeding*[Title/Abstract]) OR Enteric Feeding[Title/Abstract]) OR Diet Therapy[Title/Abstract])) |
| #36 | Search ("Enteral Nutrition"[Mesh]) OR (((((((enteral nutrition[Title/Abstract]) OR Enteral Feeding*[Title/Abstract]) OR Force Feeding*[Title/Abstract]) OR Gastric Feeding Tube*[Title/Abstract]) OR Tube Feeding*[Title/Abstract]) OR Enteric Feeding[Title/Abstract]) OR Diet Therapy[Title/Abstract]) |
| #35 | Search ((((((enteral nutrition[Title/Abstract]) OR Enteral Feeding*[Title/Abstract]) OR Force Feeding*[Title/Abstract]) OR Gastric Feeding Tube*[Title/Abstract]) OR Tube Feeding*[Title/Abstract]) OR Enteric Feeding[Title/Abstract]) OR Diet Therapy[Title/Abstract] |
| #34 | Search "Enteral Nutrition"[Mesh] |
| #32 | Search ("Nutritional Support"[Mesh]) OR (((Nutritional Support[Title/Abstract]) OR Artificial Feeding[Title/Abstract]) OR diet supplementation[Title/Abstract]) |
| #31 | Search ((Nutritional Support[Title/Abstract]) OR Artificial Feeding[Title/Abstract]) OR diet supplementation[Title/Abstract] |
| #30 | Search "Nutritional Support"[Mesh] |
| #28 | Search (((((("Arginine"[Mesh]) OR ((((L-Arginine[Title/Abstract]) OR Arginine[Title/Abstract]) OR DL-Arginine Acetate, Monohydrate[Title/Abstract]) OR DL Arginine Acetate, Monohydrate[Title/Abstract]))) OR (("Fatty Acids, Omega-3"[Mesh]) OR (((((Fatty Acids, Omega 3[Title/Abstract]) OR Omega-3 Fatty Acids[Title/Abstract]) OR omega 3 fatty acids[Title/Abstract]) OR n-3 Fatty Acids[Title/Abstract]) OR n-3 PUFA[Title/Abstract]))) OR (("Glutamine"[Mesh]) OR (((glutamine[Title/Abstract]) OR L-Glutamine[Title/Abstract]) OR D-Glutamine[Title/Abstract]))) OR (("RNA"[Mesh]) OR (((((RNA[Title/Abstract]) OR RNA Gene Product[Title/Abstract]) OR Ribonucleic Acid[Title/Abstract]) OR RNA, Non-Polyadenylated[Title/Abstract]) OR RNA, Non Polyadenylated[Title/Abstract]))) OR (((immunonutrition[Title/Abstract]) OR immunutrition[Title/Abstract]) OR immune nutritio[Title/Abstract]) |
| #27 | Search ((immunonutrition[Title/Abstract]) OR immunutrition[Title/Abstract]) OR immune nutritio[Title/Abstract] |
| #25 | Search ("RNA"[Mesh]) OR (((((RNA[Title/Abstract]) OR RNA Gene Product[Title/Abstract]) OR Ribonucleic Acid[Title/Abstract]) OR RNA, Non-Polyadenylated[Title/Abstract]) OR RNA, Non Polyadenylated[Title/Abstract]) |
| #24 | Search ((((RNA[Title/Abstract]) OR RNA Gene Product[Title/Abstract]) OR Ribonucleic Acid[Title/Abstract]) OR RNA, Non-Polyadenylated[Title/Abstract]) OR RNA, Non Polyadenylated[Title/Abstract] |
| #23 | Search "RNA"[Mesh] |
| #20 | Search ("Glutamine"[Mesh]) OR (((glutamine[Title/Abstract]) OR L-Glutamine[Title/Abstract]) OR D-Glutamine[Title/Abstract]) |
| #19 | Search ((glutamine[Title/Abstract]) OR L-Glutamine[Title/Abstract]) OR D-Glutamine[Title/Abstract] |
| #18 | Search "Glutamine"[Mesh] |
| #15 | Search ("Fatty Acids, Omega-3"[Mesh]) OR (((((Fatty Acids, Omega 3[Title/Abstract]) OR Omega-3 Fatty Acids[Title/Abstract]) OR omega 3 fatty acids[Title/Abstract]) OR n-3 Fatty Acids[Title/Abstract]) OR n-3 PUFA[Title/Abstract]) |
| #14 | Search ((((Fatty Acids, Omega 3[Title/Abstract]) OR Omega-3 Fatty Acids[Title/Abstract]) OR omega 3 fatty acids[Title/Abstract]) OR n-3 Fatty Acids[Title/Abstract]) OR n-3 PUFA[Title/Abstract] |
| #13 | Search "Fatty Acids, Omega-3"[Mesh] |
| #11 | Search ("Arginine"[Mesh]) OR ((((L-Arginine[Title/Abstract]) OR Arginine[Title/Abstract]) OR DL-Arginine Acetate, Monohydrate[Title/Abstract]) OR DL Arginine Acetate, Monohydrate[Title/Abstract]) |
| #10 | Search (((L-Arginine[Title/Abstract]) OR Arginine[Title/Abstract]) OR DL-Arginine Acetate, Monohydrate[Title/Abstract]) OR DL Arginine Acetate, Monohydrate[Title/Abstract] |
| #9 | Search "Arginine"[Mesh] |
| #6 | Search ("Stomach Neoplasms"[Mesh]) OR ((((((((gastric cancer*[Title/Abstract]) OR gastric carcinoma*[Title/Abstract]) OR gastric neoplasm*[Title/Abstract]) OR stomach neoplasm*[Title/Abstract]) OR gastric tumor*[Title/Abstract]) OR stomach cancer*[Title/Abstract]) OR stomach carcinoma*[Title/Abstract]) OR gastric tumor*[Title/Abstract]) |
| #5 | Search (((((((gastric cancer*[Title/Abstract]) OR gastric carcinoma*[Title/Abstract]) OR gastric neoplasm*[Title/Abstract]) OR stomach neoplasm*[Title/Abstract]) OR gastric tumor*[Title/Abstract]) OR stomach cancer*[Title/Abstract]) OR stomach carcinoma*[Title/Abstract]) OR gastric tumor*[Title/Abstract] |
| #4 | Search "Stomach Neoplasms"[Mesh] |

|  |
| --- |

**Embase Search Algorithm**

| **No.** | **Query** |
| --- | --- |
| #63 | #12 AND #38 AND #51 AND #62 |
| #62 | #60 OR #61 |
| #61 | random* |
| #60 | #52 OR #53 OR #54 OR #55 OR #56 OR #57 OR #58 OR #59 |
| #59 | 'phase 3 clinical trial (topic)'/exp |
| #58 | 'phase 4 clinical trial (topic)'/exp |
| #57 | 'clinical trials phase iv as topic'/exp OR 'clinical trials phase iv as topic' |
| #56 | 'clinical trials phase iii as topic'/exp OR 'clinical trials phase iii as topic' |
| #55 | 'controlled clinical trial (topic)'/exp OR 'controlled clinical trial (topic)' |
| #54 | 'controlled clinical trial'/exp OR 'controlled clinical trial' |
| #53 | 'randomized controlled trial (topic)'/exp OR 'randomized controlled trial (topic)' |
| #52 | 'randomized controlled trial'/exp OR 'randomized controlled trial' |
| #51 | #42 OR #50 |
| #50 | #43 OR #44 OR #45 OR #46 OR #47 OR #48 OR #49 |
| #49 | 'diet therapy'/exp OR 'diet therapy' |
| #48 | 'enteric feeding'/exp OR 'enteric feeding' |
| #47 | 'tube feeding'/exp OR 'tube feeding' |
| #46 | 'gastric feeding tubes'/exp OR 'gastric feeding tubes' |
| #45 | 'force feeding*' |
| #44 | 'enteral feeding'/exp OR 'enteral feeding' |
| #43 | 'enteral nutrition'/exp OR 'enteral nutrition' |
| #42 | #39 OR #40 OR #41 |
| #41 | 'diet supplementation'/exp OR 'diet supplementation' |
| #40 | 'artificial feeding'/exp OR 'artificial feeding' |
| #39 | 'nutritional support'/exp OR 'nutritional support' |
| #38 | #17 OR #23 OR #27 OR #33 OR #37 |
| #37 | #34 OR #35 OR #36 |
| #36 | 'immune nutrition' |
| #35 | 'immunonutrition' |
| #34 | immunutrition |
| #33 | #28 OR #29 OR #31 OR #32 |
| #32 | 'rna, non polyadenylated' |
| #31 | 'ribonucleic acid'/exp OR 'ribonucleic acid' |
| #29 | 'rna gene product*' |
| #28 | 'rna'/exp OR 'rna' |
| #27 | #24 OR #25 OR #26 |
| #26 | 'd glutamine' |
| #25 | 'l glutamine'/exp OR 'l glutamine' |
| #24 | 'glutamine'/exp OR 'glutamine' |
| #23 | #18 OR #19 OR #20 OR #21 OR #22 |
| #22 | 'n 3' AND pufa |
| #21 | 'n-3 fatty acid*' |
| #20 | 'omega 3 fatty acid*' |
| #19 | 'omega-3 fatty acid*' |
| #18 | 'fatty acids omega 3'/exp OR 'fatty acids omega 3' |
| #17 | #13 OR #14 OR #15 OR #16 |
| #16 | 'dl arginine acetate, monohydrate' |
| #15 | 'dl-arginine acetate, monohydrate' |
| #14 | 'arginine'/exp OR 'arginine' |
| #13 | 'l arginine'/exp OR 'l arginine' |
| #12 | #10 OR #11 |
| #11 | #2 OR #3 OR #4 OR #5 OR #6 OR #7 OR #8 OR #9 |
| #10 | 'stomach tumor'/exp |
| #9 | 'gastric tumor*' |
| #8 | 'stomach carcinoma*' |
| #7 | 'stomach cancer*' |
| #6 | 'gastric tumor*' |
| #5 | 'stomach neoplasm*' |
| #4 | 'gastric neoplasm*' |
| #3 | 'gastric carcinoma*' |
| #2 | 'gastric cancer*' |
